# First person – Sonu S. Baral

**DOI:** 10.1242/bio.052167

**Published:** 2020-04-28

## Abstract

First Person is a series of interviews with the first authors of a selection of papers published in Biology Open, helping early-career researchers promote themselves alongside their papers. Sonu S. Baral is first author on ‘Nucleolar stress in *Drosophila* neuroblasts, a model for human ribosomopathies’, published in BiO. Sonu conducted the research described in this article while a PhD student in Patrick J. DiMario's lab at Louisiana State University, Baton Rouge, LA, USA. She is now a R&D scientist at Thermo Fisher Scientific, Santa Clara, CA, USA, investigating various genetic disorders relevant to reproductive health and newborn screening.


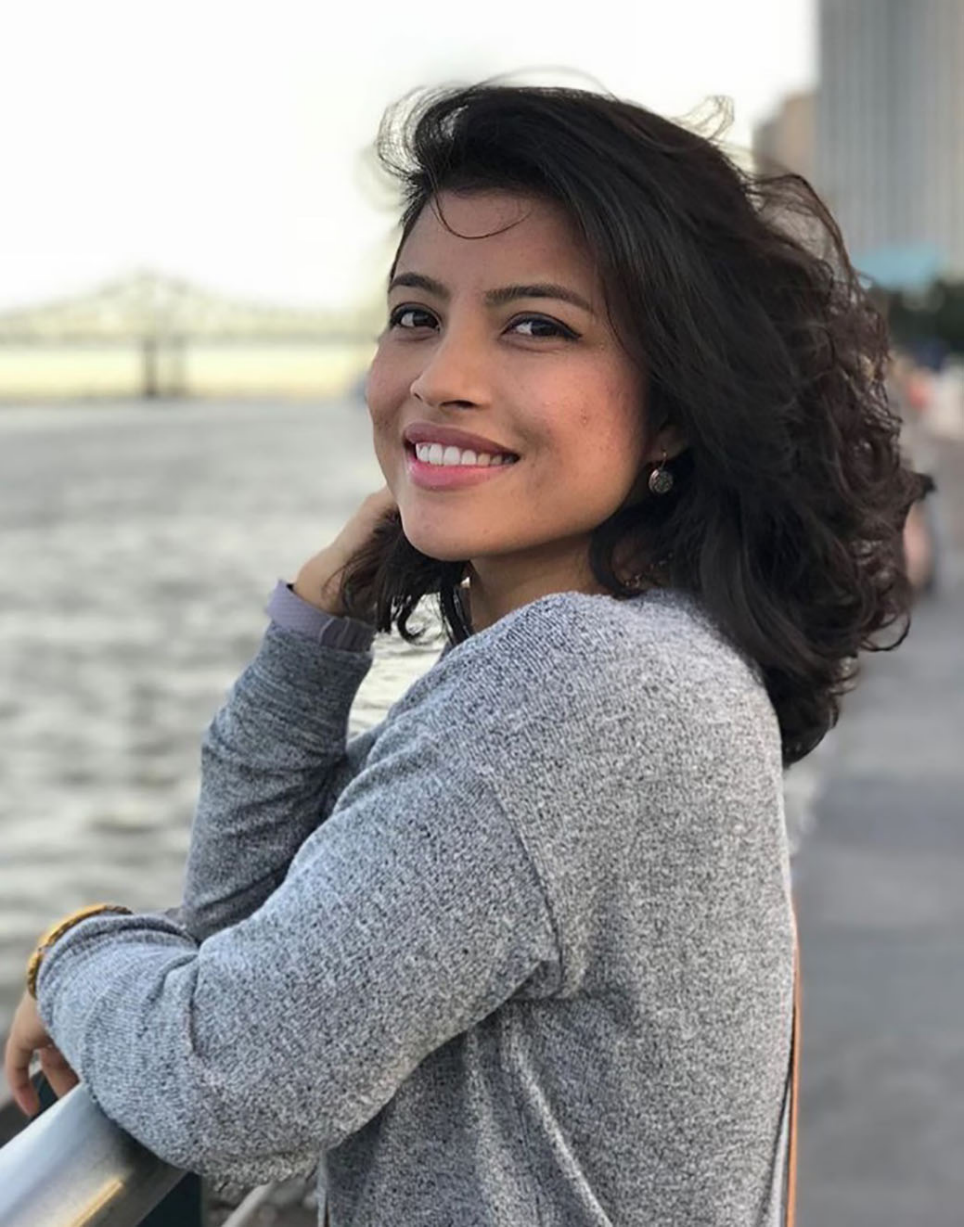


**Sonu S. Baral**

**What is your scientific background and the general focus of your lab?**

During my undergraduate studies, I participated in two biomedical summer research internships with Louisiana Biomedical Research Network. Through these internships, I was exposed to research areas involving cell and molecular biology, biochemistry, immunology and microscopy, and received hands-on training on a wide array of biology laboratory techniques. At the same time, I was working as an undergraduate researcher in an organic chemistry lab at my university where I synthesized and analyzed derivatives of hydro- and organogelators. In terms of contents and lab work, there wasn't much in common between the internships and my undergraduate research; however, what I soon realized is that I was essentially conducting scientific research in both cases, and these research experiences became crucial in my decision to join graduate school. My interest in cell and molecular biology prompted me to join the *Drosophila* (fruit fly) lab of Patrick J. DiMario at Louisiana State University as a PhD student, and I obtained my PhD degree in May 2019. The general focus of my research effort in the DiMario lab was to study nucleolar biology in the neural stem cells (neuroblasts) of *Drosophila melanogaster*. My main goal was to investigate the effects of nucleolar stress in fruit flies by depleting nucleolar ribosome-binding factors such as Nopp140 (nucleolar phosphoprotein of 140 kDa).

**How would you explain the main findings of your paper to non-scientific family and friends? **

Ribosomopathies are human genetic conditions [such as Treacher Collins Syndrome (TCS)] caused by the lack of protein-producing machineries present in cells called ribosomes. A ribosome is in itself quite complex and is made up of several components. If the genes needed to make a ribosome's structural components become non-functional, specific groups of cells become deficient of ribosomes, which disrupts normal cell functioning due to lack of proteins and eventually leads to cell death. The question that has baffled scientists studying these genetic conditions is: why are specific groups of cells, and not all cells, affected (and lost) when certain ribosome-related genes are mutated or absent? To answer this, we first generated a fruit fly strain that has a genetic condition similar to a human with TCS; we did this by disrupting a fruit fly gene called *Nopp140*, a gene essential for ribosome production and similar to the gene that is mutated in TCS individuals. Using these fruit flies with non-functional *Nopp140* genes, we showed that different stem cell types in the fruit fly brain are affected to different degrees upon loss of Nopp140 protein. We found that a group of neural stem cells called the mushroom body (MB) neuroblasts were more resilient to the effects of Nopp140 protein deficiency compared to other cell types in the brain. These resilient MB neuroblasts continued to proliferate unlike other neuroblasts (see research image). They also had detectable amounts of Nopp140 protein in the cellular space where the protein normally resides even upon complete lack of functional *Nopp140* gene. Additionally, there is some indirect evidence that suggests the presence of functional ribosomes in the MB cell lineage, which we were not expecting!

“…there is some indirect evidence that suggests the presence of functional ribosomes in the MB cell lineage, which we were not expecting!”

**Figure:**
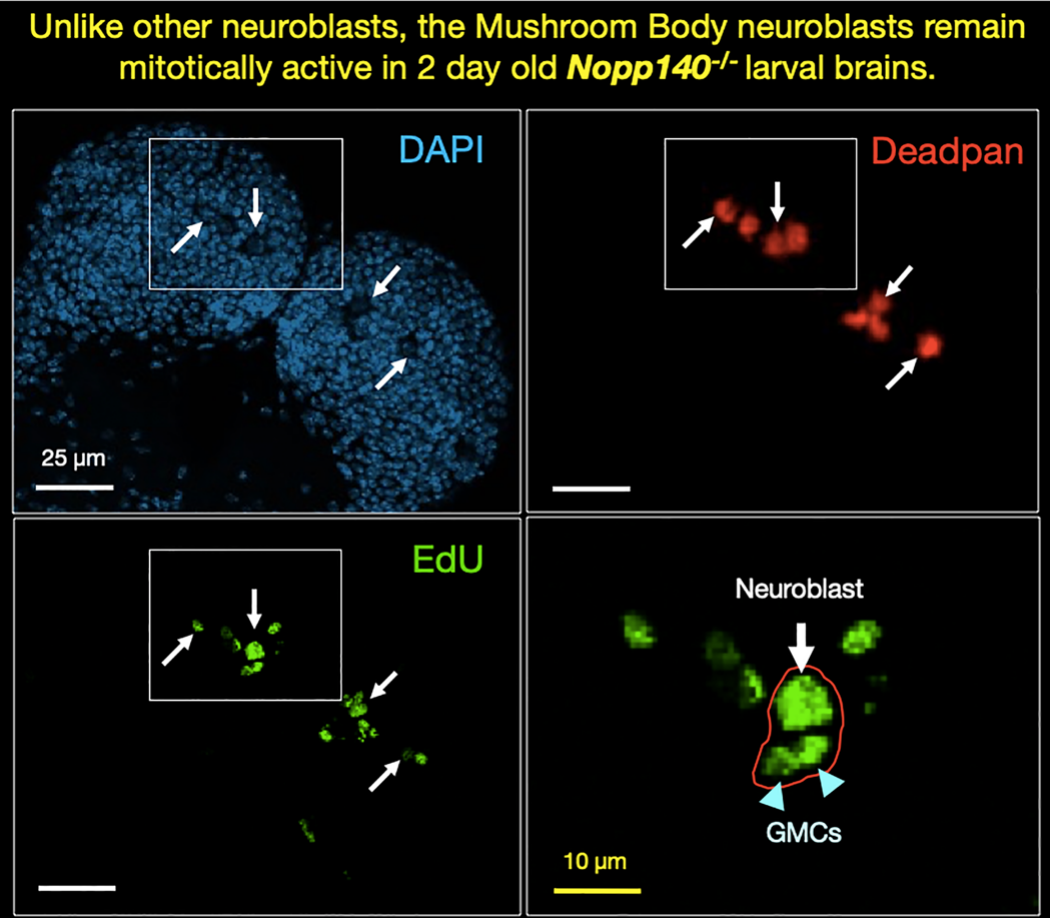
**Mitotically active Edu-labeled mushroom body neuroblasts (MB NB) in 2-day-old *Nopp140^−/−^* larval brain.** Four MB NBs per central brain lobe (arrows) were immunostained with anti-Deadpan (red), and all nuclei are counterstained with DAPI (light blue).

**What are the potential implications of these results for your field of research?**

We have generated a model system for human ribosomopathies in fruit flies that scientists can now further study and shed light onto the underlying cellular and molecular mechanisms that make specific groups of cells more or less vulnerable to the loss of ribosomes. Future work in this field may potentially lead to development of better treatments for individuals with ribosomopathies.

**What has surprised you the most while conducting your research?**

While I was aware that research requires hard work and determination, it was my PhD work that taught me to be unafraid of failures and instead accept them as learning lessons. I was surprised to see how many more experiments either fail or need to be repeated than those that give publishable results. Each one of those set-backs was another opportunity for me to exercise my patience and perseverance to push through, and as a scientist, a necessary step-forward into asking the appropriate biological question and reassessing the experiment set up.

“…it was my PhD work that taught me to be unafraid of failures and instead accept them as learning lessons.”

**What, in your opinion, are some of the greatest achievements in your field and how has this influenced your research?**

One significant achievement that has been made in the field of nucleolar biology is the that our understanding of ribosomes has evolved from indiscriminate protein-synthesizing machineries to dynamic macromolecular complexes that are involved in specialized translation of individual transcripts and even specific subsets of mRNAs. There is now direct evidence for ribosome heterogeneity (ribosomes varying in composition and function), and its implications for the regulation and expression of key gene regulatory networks are far reaching. We have observed differences in ribosome abundance among neighboring neural stem cell types within a knockout larval brain during our work on fruit fly model for ribosomopathies; we have found indirect evidence to suspect that these NSCs have heterogenous populations of ribosomes, which is on our list for further investigation. Another significant achievement in the field of neurobiology and neurodevelopment is the wealth of knowledge that exists for *Drosophila* neurogenesis making it possible to analyze developing brains at the level of individual NB lineages. This is truly fascinating and a great tool with which we can dive into the finest level of detail within a fruit fly brain as far as cellular and molecular mechanisms underlying ribosomopathies are concerned.

**What changes do you think could improve the professional lives of early-career scientists?**

One aspect that would improve the professional lives of early-career scientists is the availability of mentors who fully realize their impact on their mentees' future. Finding a mentor who understands your struggles and guides you while you're transitioning through career-phases can make a big difference. I am fortunate that I was able to conduct my research as a team with my PhD advisor who supported me every step of the way. My advisor was always present and made himself available whenever I needed his advice and expertise. Not all of my fellow graduate students received such nurturing and empowering mentorship from their mentors, which I wish wasn't the case. Additionally, lack of funding opportunities for early-career scientists have always been a big factor that deters fresh PhD graduates from pursing a post-doc research position, in my opinion. Availability of more financial support would definitely help retain PhDs in research-related careers.
